# Redox dysregulation, inflammation and mental health in adults with high risk of childhood maltreatment

**DOI:** 10.1007/s00787-026-03023-5

**Published:** 2026-05-22

**Authors:** Clara von Wendorff, Maria Meier, Daniella Dwir, Basilio Giangreco, Paul Klauser, Gizem Demirkiran, Patricia Schneider, Mickaël Das Neves, David Bürgin, Cyril Boonmann, Jörg Fegert, Marc Schmid, Vera Clemens

**Affiliations:** 1 Department for Child and Adolescent Psychiatry, Psychotherapy and Psychosomatics, University of Ulm, Steinhövelstraße 3, Ulm, 89075 Germany; 2 Child and Adolescent Psychiatric Research Department, University Psychiatric Clinics Basel (UPK), University of Basel, Basel, Switzerland; 3 Department of Psychology, University of Konstanz, Konstanz, Germany; 4 Center for Psychiatric Neuroscience, Department of Psychiatry, Centre Hospitalier Universitaire Vaudois and University of Lausanne (CHUV-UNIL), Prilly-Lausanne, Switzerland; 5 Jacobs Center for Productive Youth Development, University of Zurich, Zurich, Switzerland; 6 Department of Child and Adolescent Psychiatry, Leiden University Medical Centre (LUMC) Curium, Leiden, The Netherlands; 7 German Centre for Mental Health (DZPG), partner site Ulm, Ulm, Germany

**Keywords:** Oxidative Stress, Redox Dysregulation, Inflammation, Childhood Trauma, Childhood Adversity, Psychopathology, out-of-home care

## Abstract

Childhood maltreatment (CM) increases the risk of lifelong mental illness, yet the underlying biochemical mechanisms remain unclear. Oxidative stress (OS), alongside inflammation, may contribute to this link. This study examined whether OS mediates the link between CM and mental health in a highly vulnerable group of formerly out-of-home placed young adults and explored associations between OS and inflammatory markers. Data were collected from 131 participants (31% women, *M* age = 26.29 ± 3.5 years) with histories of youth residential care. CM was assessed using the Childhood Trauma Questionnaire (CTQ), and mental health via the Achenbach System of Empirically Based Assessment (ASEBA). Biomarkers of OS (Superoxide Dismutase [SOD], Glutathione Peroxidase/Glutathione Reductase ratio [GPx/GRed]) and inflammation (Interleukin-1 Receptor Antagonist [IL-1RA], Interleukin-6 [IL-6], Interleukin-10 [IL-10], C-Reactive Protein [CRP], Tumor Necrosis Factor [TNF𝛼]) were analyzed. Multiple regression models examined associations among CM, OS, inflammation, and mental health, adjusting for covariates. Greater CM exposure was associated with poorer mental health (*b* = 0.34, *p* < .001). Initial analyses indicated that GPx/GRed (*b =* 0.20, *p* = .04), —but not SOD— was related to CM, particularly emotional abuse (*b* = 0.21, *p* = .03). However, this association did not remain significant after adjusting for medication use, suggesting that medication may confound links between CM and redox biology. TNF𝛼 predicted current mental health problems (*b* = 0.22, *p* = .02), and OS and inflammatory markers were significantly interrelated. Neither OS nor inflammation mediated the CM–mental health relationship. These findings highlight a complex interplay between CM, OS, and inflammation in young adults with histories of residential care. They underscore the importance of considering medication effects when studying redox mechanisms and indicate that further research is needed to clarify the biological pathways linking CM to long-term mental health outcomes.

**Supplementary Information:**

The online version contains supplementary material available at https://doi.org/10.1007/s00787-026-03023-5.

## Introduction

Childhood maltreatment (CM) increases the risk for psychopathology [1, 2] and premature death [3, 4]. It exerts a profound impact on both mental and physical health [5, 6]. The precise mechanisms linking CM to the pathogenesis of mental disorders are yet not completely understood. Potential psychosocial mechanisms underlying the heightened risk for psychopathology after CM comprise disrupted attachment and social functioning, emotional dysregulation, maladaptive coping, dysfunctional cognitive schemas, and impairments in self-esteem and self-efficacy [7]. Furthermore, the dysregulation of various physiological systems, including the nervous [8], metabolic [9] and endocrine systems [10], is associated with this heightened risk for disease [11]. One system that has been repeatedly discussed to play a central role in this context is the immune system [12, 13]. The functioning of the immune system is pivotal to defending the organism from injury and pathogens and initiating healing processes.

In the context of CM, a lack of, or overshooting inflammatory responses and basal proinflammatory states may provide a gateway for psychopathology. Indeed, numerous studies have established a link between CM and subsequent inflammatory responses in both animal models [11] and humans [14–16]. For example, CM has been associated with increased levels of proinflammatory cytokines, including Interleukin-6 (IL-6), C-Reactive Protein (CRP) and Tumor Necrosis Factor alpha (TNF𝞪) [12]. These cytokines constitute mediators of the acute inflammatory response [14]. Furthermore, there is a growing body of evidence indicating a link between inflammation and a range of mental disorders, including post-traumatic stress disorder (PTSD) [17], depression [18, 19], schizophrenia [20], and anxiety disorders [17]. Moreover, inflammation has been linked to transdiagnosticrisk factors of psychopathology, such as suicidal ideation [21]. Although inflammation may mediate the association between CM and psychopathology [22], the molecular mechanisms that kindle or contribute to maintaining the inflammatory state – thereby possibly causing and furthering the negative health consequences of CM – remain poorly understood [12]. A promising avenue for further investigation in this realm is oxidative stress (OS), which is closely associated with inflammation [23, 24] and frequently observed in patients with diverse forms of psychopathology [25], including those associated with CM [26].

OS describes a state of imbalance between *pro*oxidant (e.g., reactive oxygen species [ROS]) and *anti*oxidant molecules (e.g., glutathione [GSH], superoxide dismutase [SOD]) within a cell. This imbalance can lead to cellular damage including oxidation of DNA/RNA, carbonylation of proteins and peroxidation of lipids [27]. SOD is implicated in the first line of detoxification, reducing superoxide radicals into hydrogen peroxide. Hydrogen peroxide is then reduced into water molecules through the GSH-dependent system of GSH-peroxidase (GPx)/GSH-reductase (GRed). The activation of this transcription factor leads to the release of proinflammatory cytokines, such as IL-6 and TNF𝞪, as well as the activation of immune cells [28].

In humans, OS has been related to a range of psychiatric disorders including PTSD [29], schizophrenia [26, 30–32], anxiety disorders [37], and mood disorders [33] such as depression [34], and bipolar disorder [35, 36]. Furthermore, there have been reports of close interactions between inflammation and OS in schizophrenia spectrum disorders [38]. In light of this, it may be posited that the impairing effects of CM on adult mental health may be mediated by two interconnected pathways: inflammation and OS [33, 39].

While OS is a highly promising mechanism to be studied in this context [39–42], investigations linking CM, psychopathology and OS are scarce. For example, while a growing literature on consequences of CM highlights that OS is associated with the development of physical disorders such as cardiovascular disease [43], with animal literature highlighting the causal role of OS in this relationship [44], few studies have focused on the relationships between CM, OS and psychopathology in humans [45]. The main objective of this study was therefore to test whether OS mediates the link between CM and psychopathology in a sample of young adults with previous out-of-home placements. These individuals are at an elevated risk for having experienced CM and for developing mental disorders in adulthood [46]. It was hypothesized that CM would be associated with elevated levels of psychopathology (H1) and OS (H2). Moreover, it was hypothesised that OS would be linked to psychopathology (H3). Finally, it was hypothesized that OS would act as a mediator between CM and psychopathology (H4; see Open Science Framework preregistration: https://doi.org/10.17605/OSF.IO/5J4TQ). Based on prior evidence [5], the differential effect of different types of CM, including emotional abuse, emotional neglect, physical abuse, physical neglect, sexual abuse, and different forms of psychopathology, including internalizing and externalizing problems, were explored. Finally, due to the interconnection of OS and inflammation, associations between OS and inflammation in the interplay between CM and psychopathology were explored.

## Methods

### Participants

Data was obtained from a sample of young adults who had previously participated in the *Swiss Study for Clarification and Goal-Attainment in Child Welfare and Juvenile Justice Institutions* (German: “Modellversuch Abklärung und Zielerreichung in stationären Massnahmen”, MAZ.). The data collection for the MAZ. study (baseline t0) was conducted between 2007 and 2012. In this study, a total of 592 adolescents (*M* age = 16 years; 402 male and 190 female) from 64 residential care units in Switzerland underwent a comprehensive clinical assessment comprising computer-based self- and external-assessment questionnaires and standardized clinical interviews. After approximately one year, a follow-up examination was performed using the previously used assessments (short-term follow-up t1). Approximately ten years later, between 2018 and 2020, participants who had consented to be contacted again (*n* = 511) were invited to complete online questionnaires (long-term follow-up t2, *Youth welfare Trajectories: Learning from Experience*; German: “Jugendhilfeverläufe: Aus Erfahrung Lernen” [JAEL]). In total, 231 participants completed the questionnaires and were subsequently invited to a face-to-face visit, which included several semi-structured clinical interviews and a laboratory visit. During this examination, 131 participants additionally provided blood samples (add-on studies: *Long-Term Outcomes of Childhood Adversities and Offending Behaviour* [LOCO], and *Long-term Outcomes of Childhood Adversities on DNA-Methylation* [LOC-o-met]) for the analysis of neurobiological parameters (e.g., telomere length, telomerase activity, inflammation). As compensation, participants received shopping vouchers of up to CHF 500. The study was conducted in accordance with the ethical standards outlined in the Declaration of Helsinki and was approved by the Ethics Commission of North-western Switzerland (EKNZ, Ref. 2017 − 00718).

The presented cross-sectional analyses focus on data derived at t2 and comprise the data of *N* = 131 participants (*M* age = 26.29 years, *SD* = 3.52, range = 16–38, 31% women). Further details can be found in the flowchart in the supplementary material (see Figure S1). Patterns of longitudinal dropout were examined between t0 and t2, and no significant baseline differences were found between included and excluded participants in terms of sociodemographic characteristics and psychosocial burden. See Table S1 in de supplements for more information.

### Measures

#### Sociodemographic characteristics

A computer-based self-report questionnaire was utilized to assess sociodemographic information, including age at blood draw, age at first placement, number of placements, nationality, and sex (self-reported sex assigned at birth; 1 = male, 2 = female). Potential confounding variables were assessed, included smoking status (0 = no smoker, 1 = smoker), and fasting status at blood draw (0 = not fasted, 1 = fasted), body mass index (BMI), substance consumption (cannabis, opiates, cocain, amphetamines) in the last 30 days, current medication, as well as the time between blood collection and freezing.

#### Childhood maltreatment

CM was measured retrospectively at the young adult age using the short-form of the Childhood Trauma Questionnaire (CTQ-SF). The CTQ assesses CM with 28 items, which are rated on a 5-point Likert scale. It demonstrates a good validity and reliability [47]. The dimensional *CTQ total score* (theoretical range: 25–125) was employed in the main statistical analysis, with higher scores indicating greater exposure to CM. Only 25 of the 28 items are included in the total score, because the three excluded items assess social desirability. In the exploratory analyses, the five CTQ subscales (physical neglect, emotional neglect, emotional abuse, physical abuse, and sexual abuse) were used to differentiate between the various forms of CM.

#### Mental health problems

Current mental health problems of the participants were assessed via self-report at young adult age using the Youth Self-Report (YSR) [48], or the Adult Self-Report (ASR) [49] from the Achenbach System of Empirically Based Assessment (ASEBA), depending on the age of the participants at the time of the assessment. To ensure comparability across the various questionnaire versions, the raw values were transformed to T-values based on previously published norms [50]. A value exceeding 60 indicates the presence of clinically significant symptomatology. The ASEBA total score was used as a proxy for overall mental health problems in the primary statistical analysis. It has good reliability and validity [50]. In the exploratory analysis, the ASEBA subscales for internalizing and externalizing mental health problems were employed to facilitate further differentiation between various forms of psychopathology. To descriptively assess the prevalence of mental disorders, the Structured Clinical Interview for DSM-IV (SCID-I and SCID-II) was administered [51]. Risky alcohol consumption was assessed using the Alcohol Use Disorders Identification Test (AUDIT) [52].

#### Blood collection and processing

A range of established biomarkers of redox homeostasis, including SOD, GPx, and GRed, as well as pro- and anti-inflammatory markers such as interleukin-1 receptor antagonist (IL-1RA), interleukin-6 (IL-6), interleukin-10 (IL-10), C-reactive protein (CRP), and tumor necrosis factor alpha (TNF𝞪), were evaluated. Blood samples were collected between 8:30 and 11:00 a.m. when participants were on-site for the clinical interviews. Participants were advised to fast overnight prior to the blood sampling procedure; however, 85% did not adhere to this restriction. To obtain blood plasma and serum from EDTA and CAT tubes (Becton Dickinson), samples were centrifuged at 3000 g for 10 min. Thereafter, aliquots were stored at − 80 °C until shipment and determination of IL-1RA, IL-6, IL-10, CRP, and TNF𝞪. Further details on the assays are provided below.

Peripheral blood mononuclear cells (PBMC) were obtained by a gradient density centrifugation at 800 g for 15 min. The mononuclear layer was collected and washed twice in phosphate-buffered saline (PBS). The PBMC were counted using a hemocytometer and stored as a pellet of 1 × 10^6^ at − 80 °C until shipment and determination of GPx, GRed, and SOD.

#### Oxidative stress markers

**Superoxide dismutase (SOD)** activity catalyzes the reduction of superoxide radicals into hydrogen peroxide. The formation of superoxide radicals can be attributed to the activity of xanthine oxidase, which is inhibited by SOD. Consequently, the capacity of SOD to inhibit the activity of xanthine oxidase can be assessed by measuring superoxide radical accumulation through the use of a tetrazolium salt for superoxide detection and the addition of xanthine oxidase in excess. The assay was conducted in accordance with the manufacturer’s instructions (Assay kit, Cayman chemical, Ann Arbor, MI). Briefly, 10 µl of standard curve samples and the samples (2 µg of proteins) are diluted in 200 µl of tetrazolium salt solution, in duplicates. To initiate the reaction, 20 µl of xanthine oxidase solution is added to each well and the plate is incubated for 30 min. The results are obtained by measuring the absorbance at 450 nm using a plate reader Tecan Infinite M200.The kit sensitivity is 0.005U/ml of SOD activity. The intra- and inter-assay coefficients of variation (CVs) were 2.5% and 15%, respectively.

**Glutathione peroxidase (GPx)** activity inhibits hydrogen peroxide through the utilization of GSH as a cofactor, which is subsequently converted into its oxidized form GSSG. Oxidized GSH is reduced by GSH reductase (GRed) with the use of NADPH as cofactor. Consequently, the measurement of NADPH elimination in a condition of NADPH, GSH, and GRed excess is directly proportional to GPx activity. The standard curve is constructed using a solution of NADPH at varying concentrations, with each concentration tested in duplicate. In parallel, the samples (3.5 µg of protein) to be tested and 200 µl of a phosphate buffer solution (100mM, pH 7.5) containing EDTA (0.6mM), NADPH (0.25mM), GSH (2.5mM), and GRed (0.84U/ml; Fluka) and tert-butyl hydroperoxide (TBHP, 0.8mM, Fluka) in excess are added to the wells, in triplicate. Results are obtained by measuring the absorbance at 340 nm every minute over a 5-minute period with a plate reader (Tecan Infinite M200). GPx activity is expressed as nmol/min/ul. The assay sensitivity is 0.05 nmol/min/ul. The intra- and inter-assay CVs were 2.48% and 14%, respectively.

**Glutathione reductase (GRed)** was assayed in a similar manner to GPx, utilizing the identical standard curve of NADPH, and quantifying the decline in NADPH within a solution of 200 µl phosphate buffer (100mM, pH 7.5) containing EDTA (0.6mM), oxidized glutathione (GSSG, 2.5mM) and NADPH (0.25mM), in triplicate (3.5ug of protein per sample). The results were obtained in a manner analogous to that employed for GPx, by measuring the absorbance at 340 nm every minute for 5 min, using a plate reader (Tecan Infinite M200). The GRed activity is calculated in nmol/min/µl, corresponding to the quantity of NADPH utilized for the reduction of GSSG. The assay sensitivity was 0.05 nmol/min/µl. The intra- and inter- assay CVs were 2.33% and 17% respectively.

**GPx/GRed** was calculated as the ratio between GPx and GRed, as both enzymes co-regulate the redox balance in the cell [40, 53].

#### Inflammatory markers

**Interleukin-1 receptor antagonist (IL-1RA)** (in pg/ml) was determined using a commercially available high-sensitivity ELISA (DRA00B, R&D Systems, Inc., Minneapolis, MN, USA). The kit has a lower detection limit of 18.3 pg/ml and an upper detection limit of 2000 pg/ml. Intra- and inter-assay CVs were 5.39% and 2.39%, respectively.

**Interleukin 6 (IL-6)** (in pg/ml) in plasma was determined in duplicate using a commercially available high-sensitivity ELISA (HS600C, R&D Systems, Inc., Minneapolis, MN, USA). The lower and upper detection limits of the assay were 0.09 pg/ml and 10 pg/ml, respectively. The intra-assay CV was 6.67, while the inter-assay CV was 6.09%. Two participants exhibited IL-6 levels below the detection limits, while one participant demonstrated IL-6 levels above the detection limits. Consequently, the corresponding values of the lower and upper detection limits were employed as surrogates.

**Interleukin 10 (IL-10)** (in pg/ml) was quantified using a commercially available high-sensitivity ELISA (HS100C, R&D Systems, Inc., Minneapolis, MN, USA) with a lower detection limit of 0.17 pg/ml. Due to the limited quantity of the available samples, duplicates could not be determined. A total of 23 participants (18.15%) exhibited IL-10 levels below the established detection limit. Consequently, the lower detection limit was utilized as a surrogate measurement.

**C-reactive protein (CRP)** was quantified from serum in duplicate using a commercially available high-sensitivity ELISA (EIA-3954, DRG International, Inc., USA). The intra-assay CV was 5.5%, while the inter-assay CV was 11.3%.

**Tumor Necrosis Factor alpha (TNF**𝞪**)** (in pg/ml) was determined using a commercially available high-sensitivity ELISA (HSTA00E, R&D Systems, Inc., Minneapolis, MN, USA) with a lower detection limit of 0.049 pg/ml. The intra- and inter-assay CVs were 5.19% and 7.70%, respectively. One participant exhibited levels below the established detection limit, which is why we used the lower detection limit as a surrogate.

### Statistical analysis

The statistical analyses were conducted using R (version 4.3.2) through RStudio (version 2025.09.1). Correlational analyses and model performance of regression models were conducted using the packages *car* [54] and *hmisc* [55]. The plots were created using *ggplot2* [56] and *tidyverse* [57] packages.

The assumption of normal distribution was violated for all biomarkers except SOD and TNF𝞪. Consequently, GPx/GRed, IL-1RA, IL-6, IL-10 and CRP were logarithmized prior to running the statistical models. Assome biomarkers included zero values, the application of logarithmization would have resulted in missing values. To circumvent this issue, + 0.001 was added prior to data transformation. The logarithmized values were used in the regression models, while the raw values were utilized for descriptive analyses and figures. Since GRed showed one extreme value (> 2.5 *SD*), this value was excluded from all further analyses to minimize distortions caused by the outlier.

To facilitate interpretation, all continuous variables were z-scaled. Pearson’s correlation coefficients were calculated to test whether any of the potential continuous covariates (i.e., age, BMI or time difference between blood collection and freezing) correlated significantly with the outcomes of the planned linear regression models. For dichotomic variables (i.e., sex, fasting status) ANOVAs were used to identify covariates. To account for the potential influence of substance use within the past 30 days [58, 59] and current medication on biomarker levels, ANOVA group comparisons were conducted comparing participants with and without recent use of specific substances (medication, smoking, cocaine, cannabis, amphetamines, and opiates), as well as between individuals with and without risky alcohol consumption (AUDIT > 7). In the case of a significant effect of the covariates on the biomarkers, the respective covariates were incorporated into the subsequent models to control for potential confounding effects [60]. Given the strong influence of current nutritional status on OS status and inflammation, fasting status was incorporated as a covariate in all regression models involving biomarkers [61].

To test our hypotheses, we first conducted a regression analysis to examine whether greater severity of childhood maltreatment (CTQ total score) was associated with a higher level of self-reported mental health problems (ASEBA total score). Second, to test the link between CM and OS, multiple linear regression models were run to predict SOD and GPx/GRed based on the CTQ total score. Additionally, the same analyses were run using the assessed inflammatory markers (IL-1RA, IL-6, IL-10, CRP, TNF𝞪) as well as the CTQ subscales (i.e., physical neglect, emotional neglect, emotional abuse, physical abuse, and sexual abuse). Third, multiple linear regression models were run to predict overall mental health problems (ASEBA total score) by the OS biomarkers. Exploratively, the same analyses were run using the assessed inflammatory markers and the ASEBA subscales (i.e., internalizing and externalizing mental health problems). Lastly, if the association between a specific OS or inflammatory biomarker and both CM and mental health issues reached statistical significance, a mediation model would have been run. D iagnostic plots were generated for each model, including residuals vs. fitted, normal Q–Q, scale–location (spread–location), and residuals vs. leverage plots. These plots were inspected for potential bias and model violations. For all models, the assumptions were adequately met. Finally, partial correlations between OS and inflammation markers were computed while controlling for age, sex, cannabis use, opiate use, medication use, fasting, and BMI. Multiple testing was controlled for using the Benjamini–Hochberg false discovery rate (BH FDR) correction [62], applied across all tests. Adjusted *p*-values ($\:{p}_{BH}$) were reported, with values below 0.05 considered statistically significant.

As outlined in the preregistration, we originally planned to conduct all analyses using groups with and without psychiatric disorders in addition to the ASEBA scores. As the groups were unbalanced and the group with no diagnosis was relatively small, limiting the statistical power to detect effects, these results are reported in the supplementary materials (see Table S23).

## Results

The sociodemographic characteristics of the sample are presented in Table 1. Overall, 82% of the participants had a current psychiatric diagnosis. The most prevalent diagnoses were substance use disorders (41%) and personality disorders (38%). T he Pearson’s correlation coefficients between CM, mental health problems, and the biomarkers are presented in supplementary Table S3.

**Table 1:** Sample characteristics (*N* = 131)

Variable	Mean (SD)
Age (in years)	26.29 (3.52)
Female sex (N[%])	40 (31%)
Swiss nationality (N[%])	115 (91%)
Number of placements in residential care	3.88 (3.91)
Age at first placement	11.43 (4.89)
Prevalence of current psychiatric diagnosis (SCID-I and II) (N[%])	108 (82%)
Substance use disorders	54 (41%)
Any personality disorders	50 (38%)
ADHD	30 (23%)
Mood disorders	30 (23%)
Anxiety disorders	18 (14%)
PTSD	6 (5%)
Psychotic disorders	3 (2%)
Currently taking medication (N[%])¹	53 (44%)
Substance use in the last 30 days²	
Cannabis	61 (47%)
Cocaine	16 (12%)
Amphetamine	8 (6%)
Opiate	4 (3%)
Smoking (N [%])²	90 (72%)
Risky alcohol consumption (AUDIT > 7) (N [%])²	42 (32%)
Childhood maltreatment (CTQ)²	52.10 (15.71)
Emotional neglect	15.82 (5.22)
Physical neglect	9.72 (3.34)
Emotional abuse	10.53 (5.17)
Physical abuse	8.87 (5.05)
Sexual abuse	7.17 (4.29)
Current self-reported mental health problems (ASEBA; T-values)³	55.00 (10.47)
Internalized problems	53.62 (10.99)
Externalized problems	53.86 (9.99)
GPx (nmol/min/ug)⁴	0.06 (0.02)
GRed (nmol/min/ug)⁵	0.01 (0.01)
SOD (U/ml/ug prot)⁵	0.17 (0.07)
CRP (pg/ml)	4.45 (10.08)
IL-6 (pg/ml)⁶	1.81 (1.57)
IL-10 (pg/ml)⁶	1.06 (1.14)
IL-1Ra (pg/ml)⁶	280 (218.96)
TNF𝞪 (pg/ml)⁶	0.73 (0.31)
Fasted at the time of blood draw (N [%])⁷	18 (15%)
Body Mass Index (BMI)⁸	24.84 (4.96)
Time between collection and freezing of the blood sample (in min)	19.83 (19.98)

*Notes *¹*n* = 120, ²*n* = 130, ³*n* = 125, ⁴*n* = 115, ⁵*n* = 116, ⁶*n* = 126, ⁷*n* = 124, ⁸*n* = 101. Missing data in biomarkers are mostly due to a lack of sufficient sample material. *SCID* Structured Clinical Interview for DSM-IV, *AUDIT* Alcohol Use Disorders Identification Test, *CTQ* Child Trauma Questionnaire, *ASEBA* Achenbach System of Empirically Based Assessment, *GPx* Glutathione peroxidase, *SOD* Superoxide dismutase, *GRed* Glutathione reductase, *CRP* C-reactive protein, *IL* Interleukin, *TNFa* Tumor necrosis factor alpha

In the preliminary analysis, we observed a significantly higher GPx/GRed ratio in participants reporting medication use, higher IL-1RA levels in smokers and in individuals using opiates, higher IL-6 concentrations among participants using cannabis or opiates, and lower IL-10 levels in cannabis users but higher levels in opiate users. Elevated CRP levels were found in smokers and in individuals reporting cannabis use. TNF**α** levels were increased in participants using opiates. No significant differences in biomarker concentrations were detected with respect to sex, fasting status, cocaine use, amphetamine use, or problematic alcohol use. Significant covariates were subsequently included in the main analyses. Additional details are provided in Supplementary Table S5.

### Association between childhood maltreatment and mental health problems

**Fig. 1:**
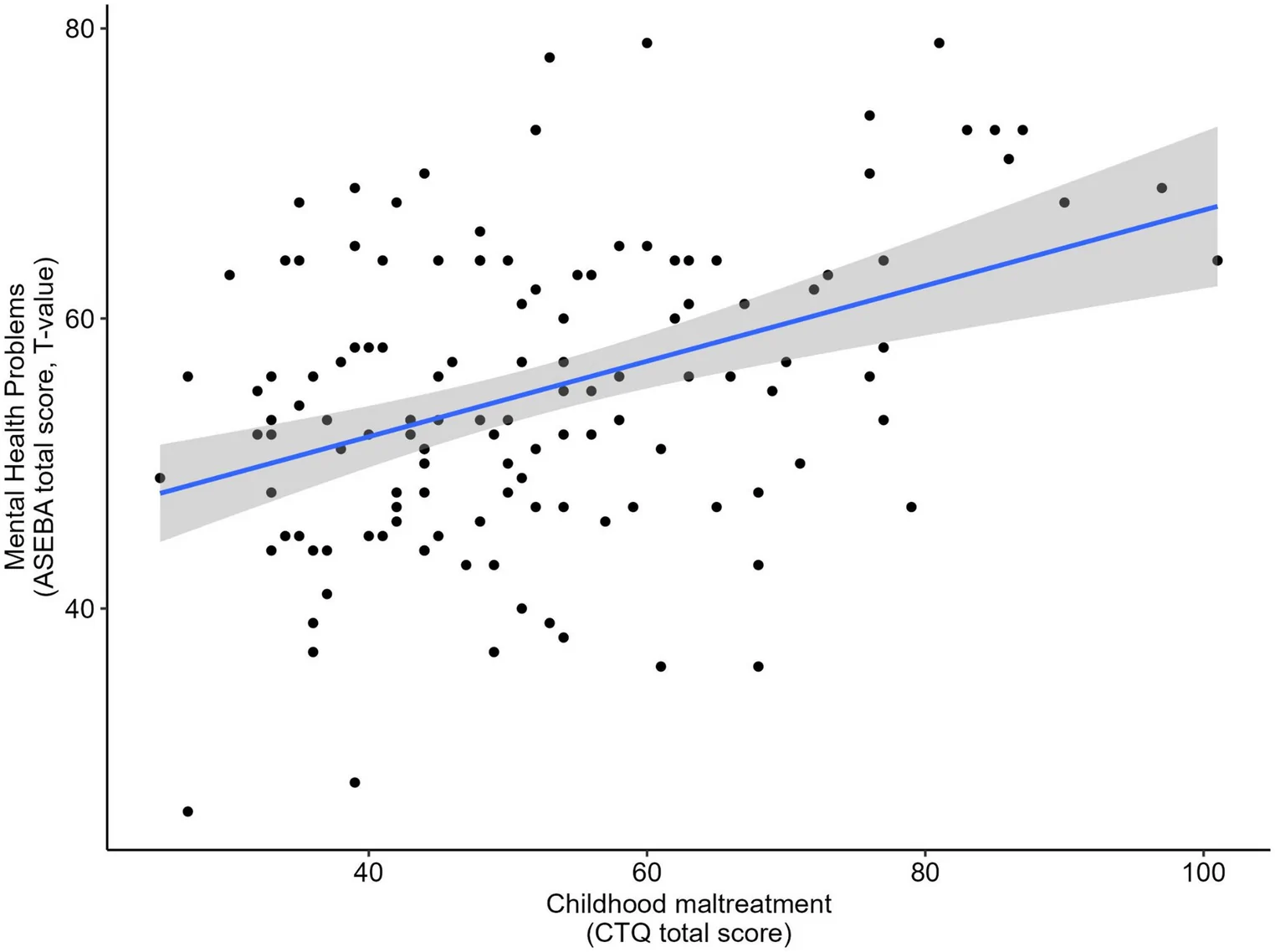
Association between childhood maltreatment (Childhood Trauma Questionnaire [CTQ] total score) and self-reported mental health problems (Achenbach System of Empirically Based Assessment [ASEBA] total score) in young adulthood

As mental health problems were signficantly associated with sex, medication use, and cannabis use, we controlled for these variables in the subsequent regression models (see Table S2 and S6 in the supplementary materials). Self-reported mental health problems (*ASEBA total score*) were significantly associated with CM (*CTQ total score*), *b* = 0.34, *p* < .001, $\:{p}_{BH\:}$< .001, when controlling for sex, *b =* 0.11, *p* = .579, $\:{p}_{BH\:}$= .579, medication use, *b =* 0.64, *p* < .001, $\:{p}_{BH\:}$< .001, and cannabis use, *b =* 0.39, *p* = .015, $\:{p}_{BH\:}$= .025. The association is illustrated in Fig. 1.

### Association between oxidative stress, inflammation and CM

**Fig. 2:**
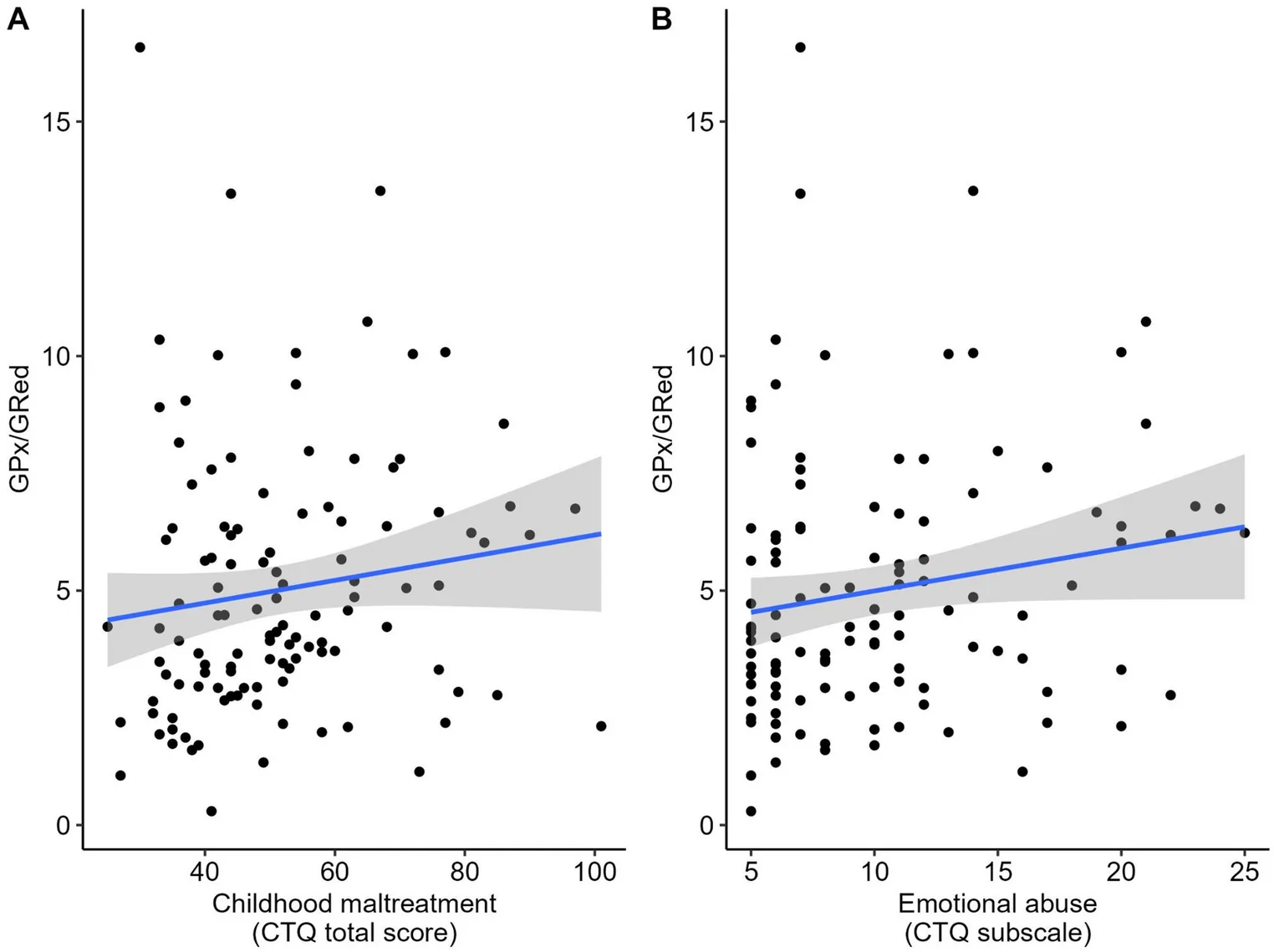
Associations between childhood maltreatment (Childhood Trauma Questionnaire [CTQ] total score and emotional abuse) and the glutathione peroxidase to glutathione reductase ratio (GPx/GRed)

Because medication use was significantly associated with the GPx/GRed ratio, both medication use and fasting status were included as covariates in the model. CM (*CTQ total score*) was a significant predictor of GPx/GRed (log), *b =* 0.20, *p* = .037*, when controlling for the effect of fasting, *b* = 0.09, *p* = .727, $\:{p}_{BH\:}$= .757, see Fig. 2A. When we entered medication use as additional covariate to the model, the effect of CM did not remain significant , *b* = 0.15, *p* = .11. After applying FDR correction for multiple comparisons, the effect of GPx/GRed on CM was also no longer statistically significant, $\:{p}_{BH\:}$= .111 (see Table S4 and S6 in the supplementary materials). CM (*CTQ total score*) was not a significant predictor of other OS and any inflammatory markers. The detailed results can be obtained from the supplements (Tables S7 and S9 to S14).

When examining the impact of distinct subtypes of CM, emotional abuse was significantly associated with GPx/GRed(log), *b =* 0.21, *p* = .03, $\:{p}_{BH\:}$= .08, when controlling for fasting status, *b =* 0.07, *p* = .78, $\:{p}_{BH\:}$= .82, (see Fig. 2B, Table S8, and S22 in the supplements). After FDR correction for multiple testing and controlling for medication use, the effect of GPx/GRed on emotional abuse was not significant anymore.

### Association between oxidative stress, inflammation and mental health problems

**Fig. 3:**
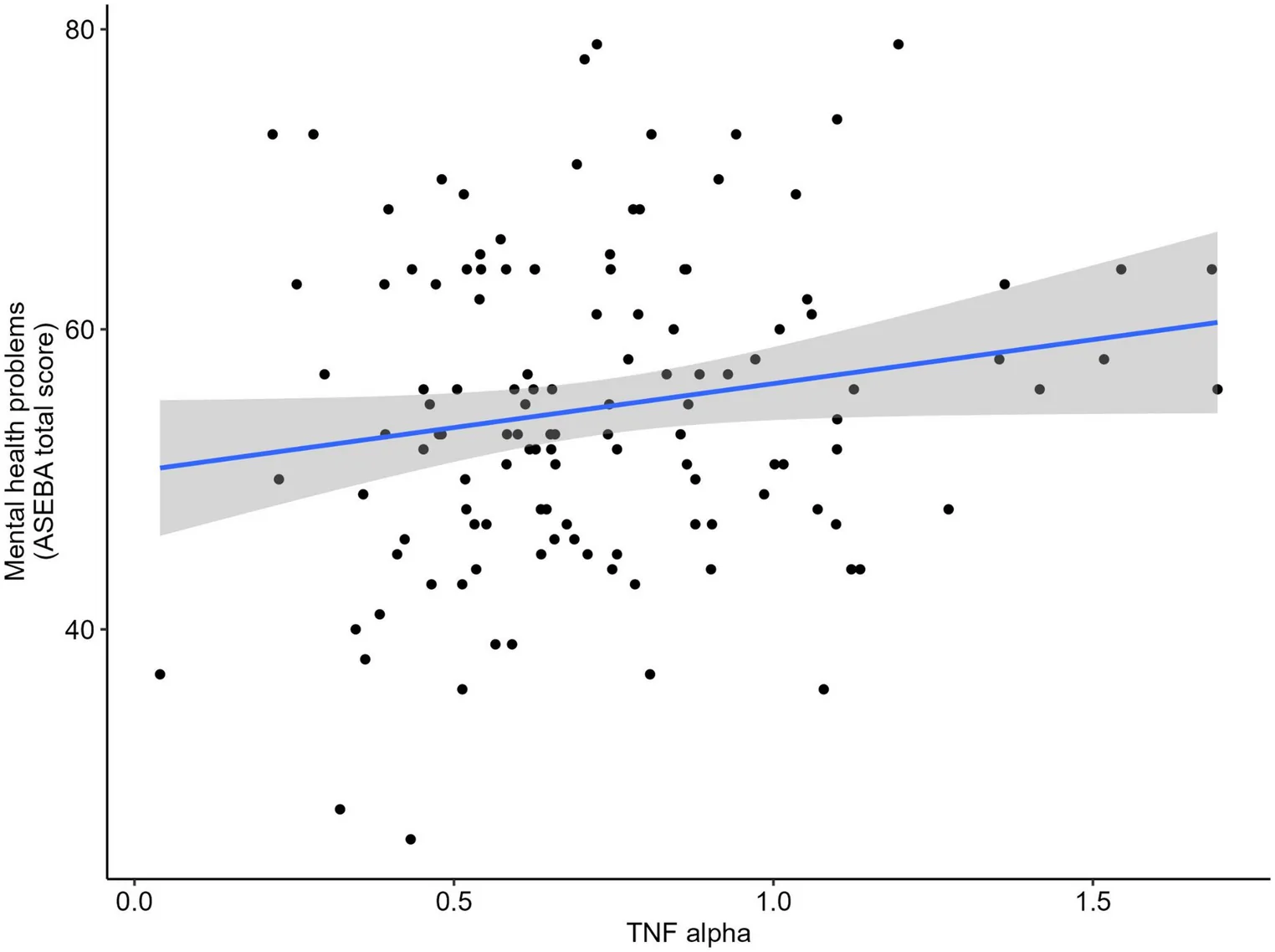
Association between tumor necrosis factor alpha (TNF𝞪) and self-reported mental health problems (Achenbach System of Empirically Based Assessment [ASEBA] total score) in adulthood

 TNF𝞪 was a significant predictor of current mental health problems (*ASEBA total score*), *b =* 0.19, *p* = .029, $\:{p}_{BH\:}$= .029, when controlling for the effect of medication use, *b =* 0.57, *p* = .002, $\:{p}_{BH\:}$= .005, sex, *b =* 0.47, *p* = .015, $\:{p}_{BH\:}$= .020, and cannabis use, *b =* 0.44, *p* = .010, $\:{p}_{BH\:}$= .017 (see Fig. 3 and Table S15 in the supplements). No additional models incorporating other OS markers (SOD, GPx/GRed) or inflammatory markers (IL-1RA, IL-6, IL-10, CRP) demonstrated statistical significance. The comprehensive results of these models can be accessed in Tables S16 to S21 in the supplementary materials.

Exploratory analyses revealed no significant relationships between externalizing or internalizing mental health problems (ASEBA subscales) and further inflammatory or OS markers (see table S21 in the supplementary materials). Given the absence of a significant association between inflammatory or redox biomarkers and CM and mental health, the planned mediation models were not calculated.

### Association between oxidative stress and inflammation

Several significant Pearson’s correlation coefficients were identified between inflammatory markers and markers of OS, as detailed in Table 2. However, after correction for multiple testing, only the association between SOD and TNF𝞪 remained significant.

**Table 2:** Correlation matrix of inflammatory and OS markers displaying Pearson’s correlations (and partial correlations controlled for age, sex, medication use, cannabis use, opiate use, BMI, and fasting in brackets)

	CRP (log)	IL-10 (log)	TNF 𝞪	IL-6 (log)	IL-1RA (log)
SOD	− 0.09 (-0.09)	0.22* (0.14)	0.25** (0.23)	− 0.16 (-0.06)	− 0.02 (-0.03)
GPx	− 0.22* (-0.06)	0.18 (0.09)	0.10 (0.02)	− 0.20* (-0.10)	− 0.06 (-0.16)
GRed	− 0.0$\:5\:$(0)	− 0.12 (-0.07)	− 0.03 (-0.03)	− 0.10 (-0.10)	− 0.01 (0.15)
GPx/GRed (log)	− 0.05 (0.07)	0.21* (-0.01)	0.13 (-0.04)	0.02 (-0.07)	0.03 (0.16)

Notes. * *p* < .05, ** *p* < .01, *** *p* < .001. For Pearson’s correlation *n* is between 114 and 116. For the partial correlation *n* = 97. After controlling for multiple testing using the Benjamini–Hochberg false discovery rate (BH FDR), only the correlation between SOD and TNF-**α** remained significant.

## Discussion

This study examined the relationship between childhood maltreatment (CM) and psychopathology, as well as their association with markers of oxidative stress (OS) and inflammation. To the best of our knowledge, this is the first study to investigate these associations using a wide range of inflammatory and OS markers in a highly vulnerable sample of young adults with previous youth residential care placements. Prior reports on the same sample indicated that higher levels of CM exposure are associated with more complex and persistent mental health problems over a ten-year period [63] and with more financial and social problems in young adulthood [64]. In alignment with prior research [1, 2, 65], elevated self-reported severity of CM was linked to a greater prevalence of mental health issues in young adulthood in the current report.

When examining the association between CM and OS, initial analyses suggested that elevated exposure to CM was associated with an increased GPx/GRed ratio, indicating an activated GSH cycle. This is consistent with findings showing that CM is associated with an altered redox state. For example, prior studies indicated that lower GPx activity and higher SOD were related to CM in adolescents [66]. Moreover, elevated GPx activity wasassociated with reduced hippocampal volume and more pronounced symptoms in individuals with early psychosis and CM [40]. Exploratory analyses provided some indication that higher levels of emotional abuse could be contributing to this association. However, the observed association between CM and the GPx/GRed ratio did not remain statistically significant after controlling for medication use. This suggests that the initially detected effect may be confounded by medication intake, which is known to influence redox-related biomarkers [67–69]. Accordingly, the patterns observed in the unadjusted analyses should be interpreted with caution. These findings highlight the importance of accounting for medication effects in studies involving redox biomarkers in highly vulnerable populations, who have a higher risk for diseases and may thus be more likely to receive pharmacological treatment as compared with other groups. 

Although the GPx/GRed ratio initially emerged as the only OS marker associated with CM, the lack of significance in adjusted models underscores the need for further research to clarify whether and how CM affects components of the GSH cycle. Previous studies have likewise reported null or inconsistent findings regarding OS markers in relation to CM [69, 70]. Replication in similarly vulnerable samples, alongside careful consideration of medication and other potential confounders will be essential to determine the robustness and specificity of CM-related alterations in redox biology. The observed specificity may partly be attributed to the characteristics of the sample, which included individuals with residential care placements only. Our results provide first evidence for differential associations between different types of trauma and certain OS markers. Previous studies have reported similar type-specific links between CM and OS in individuals with affective disorders, for example in respect to physical neglect [70], emotional neglect, emotional abuse, and sexual abuse [71]. As our sample is derived from a population of individuals who have left residential care, these exploratory, type-specific findings require replication in other highly vulnerable samples to ascertain their generalizability. It is noteworthy that the correlation between CM and OS was specific to the GPx/GRed ratio, indicating that the imbalance of the ROS system may play a pivotal role in this phenomenon. While other studies have also reported null findings [72, 73], further research, particularly on the GSH cycle, is required to elucidate the specific effects of different CM types on the various agents relevant for a balanced OS state.

Contrary to the hypothesis, no association between OS and mental health problems was found. Cumulative evidence supports heightened OS states in different patient populations, e.g., in individuals with schizophrenia [31, 74, 75] and depression [76–78]. Despite reports of redox dysregulations in stress-related disorders such as PTSD [79], which are frequently observed following CM, the evidence in this domain is heterogeneous, with some studies reporting null findings [80]. Although no inpatient sample was studied in the current report, the majority of the participants were diagnosed with at least one current psychiatric diagnosis, and around one third displayed clinically relevant mental health problems (according to ASEBA T-values > 60). The most prevalent mental disorder at the time of assessment was substance use disorder (SUD). While SUD has previously been associated with OS [81–83], substance type has been identified as a key modulator of redox dysregulation [83]. In patients with cocaine addiction, pharmacotherapy was found to have a positive impact on redox balance [84]. Given the high relevance of substance use for OS and inflammatory levels, we examined the influence of substance use on OS and inflammation and controlled for substance use where appropriate. The dependence of IL-1RA, IL-6, IL-10, CRP, and TNF𝞪 on substance use supports previous findings indicating that specific substances modulate inflammatory responses [58, 59]. Accordingly, the considerable heterogeneity within the sample as well as the lack of a control group without CM or mental illness may account for the null findings.

Although several studies have reported an association between CM and proinflammatory markers such as IL-6 and CRP [14–16], no significant correlation was found between the overall severity of CM and inflammatory markers. This inconsistency may be attributed to the fact that the effects of CM on inflammation are dependent on how CM is assessed, with discrepancies emerging between official reports and retrospective self-report [85]. Additionally, significant mediators of the effect, e.g., BMI [86] and CM types, which we considered in our analyses as well, have not been accounted for in all previous studies. Nevertheless, elevated TNF**α** levels were linked to more pronounced mental health issues, corroborating prior findings indicating a correlation between inflammation and psychopathology in individuals with PTSD [80] and depression [87].

The results of the study revealed differential associations between selected markers of OS and inflammation, as well as between these markers and subtypes of CM and mental health problems. However, these relations did not exhibit a discernible pattern, and the observed associations were relatively weak, with some not upholding the correction for multiple testing and the adjustment for covariates. Despite the proposition that OS and inflammation might mediate the link between CM and mental health problems, our data did not support a mediating role of OS or inflammation in the link between CM and mental health, a null finding also reported by others [88]. These findings are unexpected, given that CM is a well-documented stressor being linked to a range of long-lasting systemic changes [89]. Such stressors activate the hypothalamic-pituitary-adrenal (HPA) axis, the primary endocrine stress axis, which directly affects inflammation and the balance between pro- and antioxidants. This can lead to redox dysregulation and eventually to OS [90]. Given the brain’s particular vulnerability to OS due to its high concentration of oxidizable fatty acids and the accumulation of redox-active copper and iron in the context of high oxygen consumption [27], it has been proposed that severe stressors in sensitive developmental periods, such as childhood and adolescence, may exert a long-lasting effect on the redox system [41]. This could contribute to an increased risk of disease. While correlations between OS and inflammation were also evident in the current sample, with SOD being associated with TNF𝞪, future studies might investigate which of the pathways are best suited to target in mental health interventions and therapy.

The presented findings should be interpreted in light of some limitations. First, the sample of adults with previous youth residential care placement is heterogeneous with regard to the types of CM experienced and the mental health problems present at the time of assessment. To isolate the effect of CM, inclusion of individuals without CM experiences and without serious mental health issues would have been beneficial. Due to the restricted sample size, the study lacked the power to conduct more in-depth analyses of subgroups and factors that might have explained additional variance (e.g., the effect of certain types of medication). Secondly, in addition to the impact of the types of CM assessed by the CTQ, other aspects of CM, such as timing and duration, have also been demonstrated to be differentially linked to psychopathology [91]. However, this analysis did not account for these factors. Thirdly, the blood sampling protocol was not optimal for the assessment of OS markers, as the time between blood draw and freezing [92] and the fasting state at the time of sampling varied considerably between participants. Despite the samples being placed on ice and processed as soon as possible after the interview, and participants being advised to arrive in a fasted state, the recommendations could not always be complied with. This may have influenced OS markers, representing a potential confounding factor. Although we controlled for the time between blood collection and freezing, it is still possible that these factors impacted the results. At the methodological level, our study did not include direct assessments of superoxide or other reactive oxygen species production, which limits our ability to capture the full oxidative balance between radical generation and antioxidant defenses. Moreover, blood circulating antioxidant enzyme activities may not accurately reflect tissue-specific OS, particularly in brain or other organs implicated in stress and psychopathology. Lastly, the cross-sectional nature of the current approach limits the interpretation of the results in regard to causal interferences. Collecting biomarkers on more than one timepoint in future studies will be important to understand temporal dynamics of OS system dysregulations following CM across lifetime.

Despite these limitations, this study exhibits notable strengths. The sample was drawn from a highly vulnerable population with documented cases of CM, providing evidence of the suboptimal environment to which individuals were exposed to in their childhood and adolescence. Further, a range of well-characterized OS and inflammatory markers were included, which is scarce in the current literature. These comprise markers at the enzyme activity level that have been less commonly studied to date. The analyses did not provide evidence for a mediating role of OS and inflammation in the link between CM and mental health. Nevertheless, exploratory findings offered tentative indications of a potential association between redox imbalance and CM, suggesting that higher CM exposure—particularly emotional abuse—may be related to variation in ROS regulation . However, this association was no longer significant after adjusting for relevant covariates and should therefore be interpreted with caution.

Taken together, it is conceivable that a mediating relationship between CM, OS, and mental health exists, but that this relationship may have been masked by unmeasured or inadequately controlled confounders in the current study. In particular, the effects of specific medication classes and pharmacological agents, as well as patterns and types of substance use, may substantially influence redox and inflammatory markers and should be accounted for in the future. Future studies would also benefit from designs that capture temporal dynamics of OS after CM, for example through repeated biomarker assessments and longitudinal follow-up, to distinguish transient from persistent alterations. Moreover, comparing the redox status and the link to inflammation of individuals with CM and mental health problems to that of healthy individuals with and without CM exposure represents an important direction for future research. In addition, systematic investigation of other potential moderators and mediators is warranted, including the timing and duration of CM, interactions between OS and complementary neurobiological systems (e.g., the HPA axis), nutritional status, social support, and chronic somatic illness. Finally, identifying resilience factors that buffer the impact of CM on OS and mental health represents an important and clinically relevant avenue for further research.

## Supplementary Information

Below is the link to the electronic supplementary material.

Supplementary Material 1 (PDF 409 KB)

## Data Availability

Data is available upon reasonable request.
