# Supplementary material for: Redox dysregulation, inflammation and mental health in adults with high risk of childhood maltreatment
Source: Eur Child Adolesc Psychiatry. 2026 May 22;35(9):2911–24. doi: 10.1007/s00787-026-03023-5 (PMC13695656; doi:10.1007/s00787-026-03023-5)
Supplement: Supplementary file 1 [file 787_2026_3023_MOESM1_ESM.pdf]

Supplements for "*Redox dysregulation, inflammation and mental health in adults with high risk of childhood maltreatment*"

Figure S1: Flow Chart of Study Participants through the study baseline (MAZ. study), follow-up study (JAEL & JAELred study) with final sample sizes included in the current data analysis.

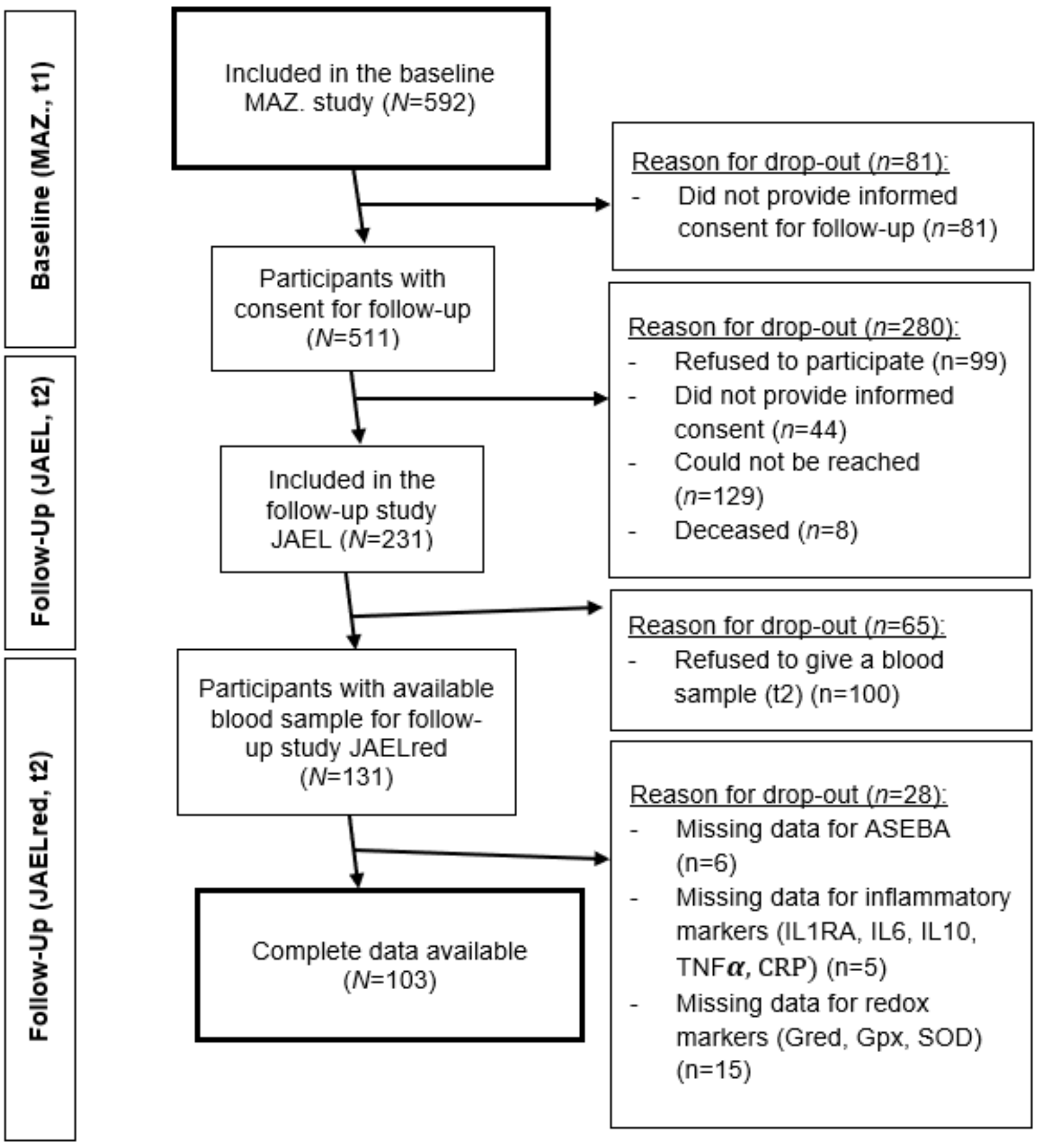

Table S1. Comparison included cases (N=115) versus non-included participants (N=482) at baseline (MAZ.) regarding psychosocial characteristics and mental health burden.

	Included	Non-included	Test-Statistic	Significance
	N (%)	N (%)		
Sex (Women)	34 (29.6)	160 (33.2)	χ^2= 0.40, *df*=1	*p*=.525
Reason for placement (t1)			χ^2= 0.37, *df* =2	*p* =.829
Civil	56 (51.9)	256 (54.1)		
Criminal	30 (27.8)	118 (24.9)		
Other	22 (20.4)	99 (20.9)		
Nationality (Swiss)	99 (90)	402 (83.4)	χ^2=2.51, *df* =1	*p* =.113
Any Mental Disorder[1]	70 (78.7)	269(68.8)	χ^2=2.935, *df* =1	*p* =.087
Any Personality Disorder[2]	18 (21.8)	90 (25.3)	χ^2=0.30, *df* =1	*p* =.587
Any Trauma[3]	46 (66.7)	178 (69.3)	χ^2=0.07, *df* =1	*p* =.790
	M (*SD*)	*M* (*SD*)		
Age	16.43 (2.49)	16.22 (2.60)	t(143.83) = -0.74	*p* =.461
Self-rep. psychopathology[4]				
Total (T-values)	61.23 (11.48)	61.02 (10.07)	t(130.34)=-0.17	*p* =.867
Internalizing (T-values)	57.84 (12.27)	57.72 (10.58)	t(129.08)=-0.09	*p* =.926
Externalizing (T-values)	61.11 (11.20)	61.01 (10.36)	t(134.51)=-0.09	*p* =.932
Childhood Maltreatment[5]	51.81 (16.22)	51.20 (16.82)	t(211.44) = -0.27	*p* =.788
Non-verbal reasoning (IQ-values)[6]	95.50 (14.91)	95.93 (14.38)	t(112.43)=0.23	*p* =.817

Notes. N=number of participants; IQ=intelligence quotient; total N of the tests performed vary between 218-592; [1] assessed with the semi-structured clinical interview Kiddie-SADS (Dölitzsch et al., 2014; Seker, Boonmann, d'Huart, et al., 2022) [2] assessed with the semi-structured clinical interview SCID-II (d'Huart, Steppan, et al., 2022; Dölitzsch et al., 2014) [3] assessed with the 'Essen Trauma-Inventory (ETI-KJ)' (Fischer et al., 2016), [4] Dimensional psychopathology was self-reported by participants at baseline with the 'Youth Self Report (YSR)' and the ‚Young Adult Self Report (YASR)' of the Achenbach scales, t-values reported (Dölitzsch, Kölch, Fegert, Schmeck, & Schmid, 2016; Seker, Bürgin, et al., 2022); [5] assessed with the Childhood Trauma Questionnaire (CTQ) sum score (Bernstein et al., 2003). [6] assessed with the Standard Progressive Matrices of Raven or the Culture Fair Intelligence Test (Habersaat et al., 2018).

Table S2: ANOVA group comparison of childhood maltreatment severity and mental health with potential dichotomic covariables

	Effect	*F* (*df1, df2*)	*p*	*mean* 1 "no" (n)	*mean* 2 "yes" (n)	*ηp²*
**Childhood maltreatment (CTQ total)**	**Male sex**	19.51 (1, 128)	<.001***	0.55 (n=39)	-0.24 (n=91)	.13
**Mental health (ASEBA total)**	**Male sex**	8.38 (1, 128)	.005**	0.38 (n=39)	-0.16 (n=91)	.06
	**Smoking**	1.16 (1, 123)	.28	-0.14 (n=35)	0.07 (n=90)	.01
	**Medication use**	11.94 (1, 117)	<.001***	-0.22 (n=74)	0.41 (n=45)	.09
	**Cocain use**	3.84 (1, 128)	.05	-0.06 (n=114)	0.45 (n=16)	.03
	**Cannabis use**	4.45 (1, 128)	.04*	-0.17 (n=69)	0.19 (n=61)	.03
	**Amphetamine use**	1.67 (1, 128)	.20	-0.03 (n=122)	0.44 (n=8)	.01
	**Opiate Use**	1.72 (1, 128)	.19	-0.02 (n=126)	0.64 (n=4)	.01
	**Critical Alcohol use**	0.21 (1, 107)	.65	-0.07 (n=88)	0.04 (n=21)	.002

Table S3: Pearson's correlations between mental health, CM, OS markers, and inflammatory markers

Biomarkers	Childhood maltreatment (CTQ)	Mental Health Problems (ASEBA)
**SOD**	.07	.11
**GPx/GRed(log)**	.18	.01
**GRed**	-.11	.07
**GPx**	.11	.06
**CRP(log)**	.04	-.03
**TNFα**	.06	.18
**IL1RA(log)**	.03	.13
**IL6(log)**	-.05	.07
**IL10(log)**	.03	.08

Note. Between n = 97 and n = 130, no correlation coefficient is statically significant.

Table S4: Pearson's correlation coefficients between the biomarkers, CTQ, ASEBA and potential continuous covariates

	**Age**	**BMI**	**Time Difference**
**SOD**	-.07	.11	.04
**GPx**	-.20*	-.09	-.03
**GRed**	-.02	.06	-.10
**GPx/GRed (log)**	-.12	-.09	.07
**CRP(log)**	.23** (BH*)	.37*** (BH***)	-.01
**TNF*α***	-.05	-.01	.16
**IL6(log)**	.27** (BH*)	.26** (BH*)	-.06
**IL10(log)**	-.04	.03	.06
**IL1RA(log)**	.05	.41*** (BH***)	.22* (BH n.s.)
**ASEBA total**	-.16	.06	.17
**CTQ total**	-.08	.01	.22* (BH n.s.)

Note. *** $p < .001$, ** $p < .01$, * $p < .05$, BMI= Body mass index, BH = significance after correction for multiple testing using the Benjamini–Hochberg false discovery rate (FDR) procedure. Between $n = 86$ and $n = 131$.

Table S5: ANOVA-based group comparison of oxidative stress and inflammation Biomarkers with dichotomous covariates

Biomarker	Effect	F (df1, df2)	*p*	*mean* 1 "no" (n)	*mean* 2 "yes" (n)	ηp^2
**SOD**	**Male sex**	0.17 (1, 114)	.68	-.06 (n=35)	0.03 (n=81)	.001
	**Fasting**	0.47 (1, 108)	.49	-0.03 (n=93)	0.14 (n=18)	.004
	**Smoking**	0.06 (1,109)	.80	-0.04 (n=33)	0.01 (n=78)	.001
	**Current medication**	1.14 (1,108)	.29	-0.08 (n=67)	0.12 (n=43)	.01
	**Cocaine use**	0 (1, 113)	.99	0 (n=99)	0 (n=16)	0
	**Cannabis use**	3.14 (1, 113)	.08	0.15 (n=61)	-0.17 (n=54)	.03
	**Amphetamine use**	0 .28 (1, 113)	.60	0.01 (n=108)	-0.19 (n=7)	.002
	**Opiate use**	0.92 (1, 113)	.34	-0.01 (n=112)	0.55 (n=3)	.01
	**Critical alcohol use**	1.11 (1, 95)	.30	-0.02 (n=78)	0.25 (n=19)	.01
**GPx/GRed**	**Male sex**	2.06 (1, 113)	.15	-0.09 (n=80)	0.2 (n=35)	.02
	**Fasting**	0.02 (1, 107)	.89	-0.03 (n=91)	0.01 (n=18)	<.001
	**Smoking**	0.42 (1, 108)	.52	-0.12 (n=33	0.02 (n=77)	.004
	**Current medication**	6.41 (1, 107)	.01*	-0.21 (n=61)	0.27 (n=48)	.06
	**Cocaine use**	0.28 (1, 112)	.60	0.02 (n=98)	-0.12 (n=16)	.002
	**Cannabis use**	2.04 (1, 112)	.16	0.13 (n=61)	-0.14 (n=53)	.02
	**Amphetamine use**	2.32 (1, 112)	.13	0.04 (n=107)	-0.55 (n=7)	0
	**Opiate use**	0.27 (1, 112)	.61	-0.05 (n=111)	0.30 (n=3)	.002

	**Critical alcohol use**	0.32 (1, 94)	.57	-0.02 (n=78)	-0.17 (n=18)	.003
**IL1RA**	**Male sex**	0.38 (1, 124)	.54	-0.04 (n=87)	0.08 (n=39)	.003
	**Fasting**	1.12 (1, 118)	.29	-0.01 (n=103)	0.03 (n=17)	.01
	**Smoking**	4.33 (1, 119)	.04*	-0.30 (n=34)	0.11 (n=87)	.04
	**Current medication**	2.76 (1,115)	.10	-0.14 (n=66)	0.15 (n=51)	.02
	**Cocaine use**	0 (1, 123)	.98	0 (n=110)	0.01 (n=15)	0
	**Cannabis use**	0.04 (1, 123)	.84	-0.01 (n=69)	0.02 (n=56)	0
	**Amphetamine use**	0.53 (1, 123)	.47	-0.01 (n=117)	0.25 (n=8)	.004
	**Opiate use**	6.71 (1, 123)	.01*	-0.04 (n=121)	1.25 (n=4)	.05
	**Critical alcohol use**	0.04 (1, 103)	.85	0.03 (n=85)	0.08 (n=20)	<.001
**IL6**	**Male sex**	1.47 (1, 124)	.23	0.07 (n=87)	-0.16 (n=39)	.01
	**Fasting**	1.22 (1, 118)	.27	0.04 (n=103)	-0.24 (n=17)	.01
	**Smoking**	3.33 (1, 119)	.07	0.25 (n=34)	-0.12 (n=87)	.03
	**Current medication**	0.001 (1, 115)	.97	0.02 (n=66)	0.01 (n=51)	0
	**Cocaine use**	2.64 (1, 123)	.11	-0.04 (n=110)	0.40 (n=15)	.02
	**Cannabis use**	13.36 (1, 123)	<.001***	-0.27 (n=69)	0.36 (n=56)	.10
	**Amphetamine use**	0.02 (1, 123)	.88	0.01 (n=117)	-0.04 (n=8)	0
	**Opiate use**	7.41 (1, 123)	.01**	-0.3 (n=121)	1.31 (n=4)	.06
	**Critical alcohol use**	2.51 (1, 103)	.12	0.14 (n=85)	-0.22 (n=20)	.02

**IL10**	**Male sex**	0.01 (1, 124)	.94	0 (n=87)	0.01 (n=39)	<.001
	**Fasting**	0.10 (1, 118)	.75	-0.02 (n=103)	0.06 (n=17)	.001
	**Smoking**	3.33 (1, 119)	.07	0.25 (n=34)	-0.12 (n=87)	.03
	**Current medication**	2.91 (1, 115)	.09	-00.16 (n=66)	0.16 (n=51)	.02
	**Cocaine use**	1.11 (1, 123)	.29	0.03 (n=110)	-0.26 (n=15)	.01
	**Cannabis use**	6.72 (1, 123)	.01*	0.20 (n=69)	-0.26 (n=56)	.05
	**Amphetamine use**	0.63 (1, 123)	.43	-0.03 (n=117)	0.27 (n=8)	.01
	**Opiate use**	4.82 (1, 123)	.03*	-0.04 (n=121)	1.06 (n=4)	.04
	**Critical alcohol use**	1.10 (1, 103)	.30	0.06 (n=85)	-0.21 (n=20)	.01
**CRP**	**Male sex**	1.13 (1, 129)	.30	.06 (n=91)	-0.14 (n=40)	.01
	**Fasting**	0.28 (1, 122)	.60	0.03 (n=106)	-0.1 (n=18)	.002
	**Smoking**	10.66 (1, 123)	.001**	-0.45 (n=35)	0.18 (n=90)	.08
	**Current medication**	0.24 (1, 118)	.62	-0.05 (n=67)	0.04 (n=53)	.002
	**Cocaine use**	1.83 (1, 128)	.18	-0.04 (n=114)	0.32 (n=16)	.01
	**Cannabis use**	4.19 (1, 128)	.04*	-0.16 (n=69)	0.19 (n=61)	.03
	**Amphetamine use**	0.11 (1, 128)	.74	0 (n=122)	0.12 (n=8)	0
	**Opiate use**	3.06 (1, 128)	.08	-0.02 (n=126)	0.86 (n=4)	.02
	**Critical alcohol use**	0.01 (1, 108)	.93	0.06 (n=89)	0.04 (n=21)	<.001

**TNF alpha**	**Male sex**	1.44 (1, 124)	.23	0.07 (n=87)	-0.16 (n=39)	.01
	**Fasting**	0.02 (1, 118)	.88	-0.01 (n=103)	0.03 (n=17)	<.001
	**Smoking**	0.20 (1, 119)	.66	0.06 (n=34)	-0.04 (n=87)	.002
	**Current medication**	3.19 (1, 115)	.08	-0.17 (n=66)	0.15 (n=51)	.03
	**Cocaine use**	0.47 (1, 123)	.49	-0.02 (n=110)	0.17 (n=15)	.004
	**Cannabis use**	0.03 (1, 123)	.88	0.02 (n=69)	-0.01 (n=56)	0
	**Amphetamine use**	0.43 (1, 123)	.52	-0.01 (n=117)	0.23 (n=8)	.003
	**Opiate use**	6.62 (1, 123)	.01*	-0.04 (n=121)	1.25 (n=4)	.05
	**Critical alcohol use**	0.63 (1, 103)	.43	-0.08 (n=85)	0.1 (n=20)	.01

Note: The substance use was measured as use in the last 30 days. All means are z-standardizes.

Table S6: Results of the regression model in which we predicted mental health problems (ASEBA total score) by CM (CTQ total score).

**Predictor**	***b***	***p***	***p (BH)***
Intercept	-0.59	.033*	.041*
CTQ total score	0.34	<.001***	<.001***
sex	0.11	.579	.579
Cannabis use [1]	0.39	.015*	.025*
Medication [1]	0.64	<.001***	.001***

Note. [1] 1 = yes, 0 = no, *** $p < .001$, $n = 96$, p (BH) = p-values adjusted for multiple testing using the Benjamini–Hochberg false discovery rate (FDR) procedure.

Table S7: Results of the regression model in which we predicted GPx/GRed (log) by CM (CTQ total score).

**Predictor**	**b**	***p***	***p (BH)***
Intercept	-0.22	.130	.174
CTQ total	0.15	.110	.174
Medication [1]	0.42	.038*	.154
Fasting [1]	0.15	.571	.571

Note. [1] 1 = yes, 0 = no, * $p < .05$, $n = 87$, *p* (BH) = *p*-values adjusted for multiple testing using the Benjamini–Hochberg false discovery rate (FDR) procedure.

Table S8: Results of the regression model in which we predicted SOD by CM (CTQ total score).

**Predictor**	**b**	***p***	***p (BH)***
Intercept	-0.03	.744	.744
CTQ total	0.04	.632	.744
Fasting [1]	0.19	.468	.744

Note. [1] 1 = yes, 0 = no, $n = 114$, *p* (BH) = *p*-values adjusted for multiple testing using the Benjamini–Hochberg false discovery rate (FDR) procedure.

Table S9: Results of the regression model in which we predicted GPx/GRed (log) by emotional abuse (CTQ subscale).

**Predictor**	**b**	***p***	***p (BH)***
Intercept	-0.20	.152	.203
CTQ emotional abuse	0.17	.075	.150
Medication [1]	0.40	.044*	.150
Fasting [1]	0.13	.612	.612

Note. [1] 1 = yes, 0 = no, * $p < .05$, $n = 87$, *p* (BH) = *p*-values adjusted for multiple testing using the Benjamini–Hochberg false discovery rate (FDR) procedure.

Table S10: Results of the regression model in which we predicted IL1RA(log) by CM (CTQ total score).

**Predictor**	**b**	***p***	***p (BH)***
Intercept	-0.29	.108	.377
CTQ total	0.02	.849	.849
BMI	0.36	<.001***	.002**
Time difference	0.04	.723	.844
Fasting [1]	0.31	.284	.397
Smoking [1]	0.26	.205	.397
Opiate use [1]	0.65	.227	.397

Note. [1] 1 = yes, 0 = no, *** $p < .001$, $n = 96$, *p* (BH) = *p*-values adjusted for multiple testing using the Benjamini–Hochberg false discovery rate (FDR) procedure.

Table S11: Results of the regression model in which we predicted IL-6(log) by CM (CTQ total score)

**Predictor**	**b**	***p***	***p (BH)***
Intercept	-0.43	.003**	.009**
CTQ total	-0.04	.566	.583
Age	0.04	.582	.582
Cannabis use	0.75	<.001***	<.001***
Opiate use	0.80	.066	.133
BMI	0.27	<.001***	.003**
Fasting [1]	-0.28	.249	.332

Note. [1] 1 = yes, 0 = no, * $p < .05$, $n = 96$, for substance use 0 = “no use”, 1 = “use of the substance within last 30 days” , *p* (BH) = *p*-values adjusted for multiple testing using the Benjamini–Hochberg false discovery rate (FDR) procedure.

Table S12: Results of the regression model in which we predicted IL-10(log) by CM (CTQ total score)

**Predictor**	**b**	***p***	***p (BH)***
Intercept	0.15	.230	.383
CTQ total	0.03	.751	.751
Cannabis use	-0.56	.002**	.010**
Opiate use	1.48	.004**	.010**
Fasting [1]	0.22	.388	.484

Note. [1] 1 = yes, 0 = no, n = 124, p (BH) = p-values adjusted for multiple testing using the Benjamini–Hochberg false discovery rate (FDR) procedure.

Table S13: Results of the regression model in which we predicted CRP(log) by CM (CTQ total score).

**Predictor**	**b**	***p***	***p (BH)***
Intercept	-0.49	.006**	.022*
CTQ total	0.03	.689	.689
Age	0.06	.508	.593
Cannabis use	0.49	.013*	.029*
BMI	0.36	<.001***	.001**
Fasting [1]	-0.21	.461	.593

Note. [1] 1 = yes, 0 = no, *** $p < .001$, n = 99, for substance use 0 = “no use”, 1 = “use of the substance within last 30 days” , p (BH) = p-values adjusted for multiple testing using the Benjamini–Hochberg false discovery rate (FDR) procedure.

Table S14: Results of the regression model in which we predicted TNF$\boldsymbol{\alpha}$ by CM (CTQ total score).

**Predictor**	**b**	***p***	***p (BH)***
Intercept	-0.06	.535	.671
CTQ total	0.08	.382	.671
Opiate use	1.31	.011*	.044*
Fasting [1]	0.11	.671	.671

Note. [1] 1 = yes, 0 = no, n = 124, p (BH) = p-values adjusted for multiple testing using the Benjamini–Hochberg false discovery rate (FDR) procedure.

Table S15: Results of the regression model in which we predicted mental health problems (ASEBA total score) by TNF$\boldsymbol{\alpha}$.

**Predictor**	**b**	***p***	***p (BH)***
Intercept	-1.05	<.001***	.001***
TNF$\boldsymbol{\alpha}$	0.19	.029*	.029*
Sex	0.47	.015**	.020*
Medication [1]	0.57	.002**	.005**
Cannabis use [1]	0.44	.010*	.017*

Note. [1] 1 = yes, 0 = no, * p < .05, ** p <.01, n = 94, p (BH) = p-values adjusted for multiple testing using the Benjamini–Hochberg false discovery rate (FDR) procedure.

Table S16: Results of the regression model in which we predicted mental health problems (ASEBA total score) by SOD.

**Predictor**	**b**	***p***	***p (BH)***
Intercept	-1.07	<.001***	.001**
SOD	0.14	.108	.108
Sex	0.44	.033*	.042*
Medication [1]	0.76	.001***	.001**
Cannabis use [1]	0.53	.004**	.010**

Note. [1] 1 = yes, 0 = no, * p < .05, ** p <.01, n = 87, p (BH) = p-values adjusted for multiple testing using the Benjamini–Hochberg false discovery rate (FDR) procedure.

TableS17: Results of the regression model in which we predicted mental health problems (ASEBA total score) by GPx/GRed (log).

**Predictor**	**b**	***p***	***p (BH)***
Intercept	-1.01	<.001***	.001**
GPx/GRed(log)	-0.04	.681	.681
Sex	0.40	.054	.066
Medication [1]	0.70	<.001***	.002**
Cannabis use [1]	0.49	.008**	.013*

Note. [1] 1 = yes, 0 = no, * p < .05, ** p <.01, n = 87, p (BH) = p-values adjusted for multiple testing using the Benjamini–Hochberg false discovery rate (FDR) procedure.

Table S18: Results of the regression model in which we predicted mental health problems (ASEBA total score) by IL1RA(log).

**Predictor**	**b**	***p***	***p (BH)***
Intercept	-0.967	<.001***	.002**
IL1RA(log)	0.10	.269	.269
Sex	0.40	.042*	.053*
Medication [1]	0.63	<.001***	.002**
Cannabis use [1]	0.41	.019*	.032*

Note. [1] 1 = yes, 0 = no, ** p <.01, n = 94, p (BH) = p-values adjusted for multiple testing using the Benjamini–Hochberg false discovery rate (FDR) procedure.

Table S19: Results of the regression model in which we predicted mental health problems (ASEBA total score) by IL-6(log).

**Predictor**	**b**	***p***	***p (BH)***
Intercept	-1.00	<.001***	.001**
IL6(log)	0.04	.622	.622
Sex	0.41	.035**	.044*
Medication [1]	0.65	<.001***	.001**
Cannabis use [1]	0.39	.031*	.044*

Note. [1] 1 = yes, 0 = no, ** p <.01, n = 94, p (BH) = p-values adjusted for multiple testing using the Benjamini–Hochberg false discovery rate (FDR) procedure.

Table S20: Results of the regression model in which we predicted mental health problems (ASEBA total score) by IL-10(log).

**Predictor**	**b**	***p***	***p (BH)***
Intercept	-1.03	<.001**	.002**
IL10(log)	0.10	.280	.280
Sex	0.43	.029*	.036*
Medication [1]	0.62	<.001	.002**
Cannabis use [1]	0.46	.010**	.016*

Note. [1] 1 = yes, 0 = no, * p < .05, ** p <.01, n = 94, p (BH) = p-values adjusted for multiple testing using the Benjamini–Hochberg false discovery rate (FDR) procedure.

Table S21: Results of the regression model in which we predicted mental health problems (ASEBA total score) by CRP(log).

**Predictor**	**b**	***p***	***p (BH)***
Intercept	-0.95	<.001***	.002**
CRP(log)	-0.07	.440	.440
Sex	0.347	.074	.092
Medication [1]	0.69	<.001***	.001***
Cannabis use [1]	0.43	.014*	.024*

Note. [1] 1 = yes, 0 = no, * $p < .05$, ** $p < .01$, $n = 96$, p (BH) = p-values adjusted for multiple testing using the Benjamini–Hochberg false discovery rate (FDR) procedure.

Table S22: Pearson's correlations between biomarkers and subtypes of CM (CTQ subscales) and mental health problems (ASEBA subscales)

Biomarkers	Emotional abuse (CTQ)	Physical abuse (CTQ)	Physical neglect (CTQ)	Emotional neglect (CTQ)	Sexual abuse (CTQ)	Internalizing mental health problems (ASEBA)	Externalizing mental health problems (ASEBA)
SOD	.09	.03	-.02	-.01	.14	.09	.03
GPx/GRed (log)	.19* (BH n.s.)	.11	.02	.14	.14	.10	-.06
GRed(log)	-.17	-.02	0	-.06	-.09	-.03	.10
GPx	.08	.02	-.01	.14	.10	.16	.04
CRP(log)	-.02	.11	.05	.06	-.08	-.14	-.07
TNFα	.01	-.01	.04	.11	.07	.13	.11
IL1RA(log)	.10	-.02	-.01	0	.10	.09	.08
IL6(log)	-.06	-.02	.05	-.07	-.06	-.01	-.01
IL10(log)	.07	-.02	-.13	.04	.08	.03	-.03

Note. n = between 115 and 130, * p<.05., p-value no longer significant after Benjamini–Hochberg FDR correction for multiple testing.

Table S23: Differences in OS and inflammation level between participants with and without any psychiatric diagnosis (HITOP).

	Without psychiatric diagnosis		With any psychiatric diagnosis					
	M	*SD*	*M*	*SD*	*df*	*t*	*p*	*Cohen's d*
SOD	0.34	1.03	-0.07	1	106	1.49	.14	.41
GPx/GRed(log)	-0.24	1.02	-0.03	0.98	106	-0.71	.48	.20
IL1-R	-0.21	0.97	-.01	0.98	116	-0.82	.42	.20
IL6	-0.43	0.7	0.09	1.02	116	-2.13	.04*	.53
IL10	0.05	0.93	-0.05	1.03	116	0.37	.71	.09
CRP	-0.12	1.06	0.04	0.95	121	-0.66	.51	.16
TNF	-0.07	0.96	-0.06	0.96	116	-0.05	.96	.01

Note. All biomarkers were z-standardized.
